# Correlated production and consumption of chloromethane in the *Arabidopsis thaliana* phyllosphere

**DOI:** 10.1038/s41598-017-17421-y

**Published:** 2017-12-14

**Authors:** Muhammad Farhan Ul Haque, Ludovic Besaury, Thierry Nadalig, Françoise Bringel, Jérôme Mutterer, Hubert Schaller, Stéphane Vuilleumier

**Affiliations:** 1 Université de Strasbourg, CNRS, GMGM UMR 7156, Department of Microbiology, Genomics and the Environment, Strasbourg, France; 2 Institut de Biologie Moléculaire des Plantes, UPR 2357 CNRS Strasbourg, France; 3 Present Address: School of Environmental Sciences, University of East Anglia, Norwich, UK; 4 Present Address: Université de Reims Champagne Ardenne, INRA, FARE UMR, A614 Reims, France

## Abstract

Chloromethane (CH_3_Cl) is a toxic gas mainly produced naturally, in particular by plants, and its emissions contribute to ozone destruction in the stratosphere. Conversely, CH_3_Cl can be degraded and used as the sole carbon and energy source by specialised methylotrophic bacteria, isolated from a variety of environments including the phyllosphere, i.e. the aerial parts of vegetation. The potential role of phyllospheric CH_3_Cl-degrading bacteria as a filter for plant emissions of CH_3_Cl was investigated using variants of *Arabidopsis thaliana* with low, wild-type and high expression of *HOL1* methyltransferase previously shown to be responsible for most of CH_3_Cl emissions by *A*. *thaliana*. Presence and expression of the bacterial chloromethane dehalogenase *cmuA* gene in the *A*. *thaliana* phyllosphere correlated with *HOL1* genotype, as shown by qPCR and RT-qPCR. Production of CH_3_Cl by *A*. *thaliana* paralleled *HOL1* expression, as assessed by a fluorescence-based bioreporter. The relation between plant production of CH_3_Cl and relative abundance of CH_3_Cl-degrading bacteria in the phyllosphere suggests that CH_3_Cl-degrading bacteria co-determine the extent of plant emissions of CH_3_Cl to the atmosphere.

## Introduction

The phyllosphere, defined as the aerial part of plants and especially leaves, is a crucial if perhaps still overlooked ecosystem, with leaf surfaces estimated to total 1 × 10^9^ km², i.e. twice the land surface area. The phyllosphere represents a major habitat for micro-organisms. Bacteria, in particular, are present at 10^6^–10^7^ cells per cm² of leaf surface, as determined by both culture-dependent and culture-independent studies^1^.

Availability of carbon is an important factor for microbial colonisation of the phyllosphere^2^. Methanol (global estimates of 100 Tg /y for plant emissions^3^) and photosynthates such as fructose, sucrose and glucose, are prominent carbon sources on leaves^2^. Methanol represents a privileged growth substrate for methylotrophic bacteria, and such micro-organisms, in particular from the Alphaproteobacterial genus *Methylobacterium*, are dominant in the phyllosphere^4^.

Chloromethane is another volatile one-carbon compound produced by plant leaves^5^, with global production currently estimated at approximately 1–5 Tg/y^6^. Although quantitatively far less important than methanol, chloromethane has environmental significance as the most abundant halogenated hydrocarbon in the atmosphere (~550 ppt), and because it is responsible for about 15% of chlorine-catalysed destruction of stratospheric ozone^6^. The best characterised mechanism for chloromethane production involves a methyltransferase-catalysed reaction, first demonstrated in *Brassica oleracea* ^7^. In *Arabidopsis thaliana*, the methyltransferase gene *HOL1* (Harmless to Ozone Layer) was identified by a knock-out mutation leading to loss of chloromethane production^8^. A halide methyltransferase gene homolog of *HOL1* in *Raphanus sativus* (daikon radish, another member of *Brassicaceae* family) was since also shown to be involved in methyl halide emissions^9^.

Some specialised methylotrophic bacteria utilise chloromethane as their only source of carbon and energy for growth^10,11^, and may thus represent natural sinks for chloromethane emissions. In this context, methylotrophic chloromethane-degrading strains from the phyllosphere of *A*. *thaliana* were isolated^12^. These isolates feature the same chloromethane utilisation pathway previously characterised in detail for *Methylobacterium extorquens* CM4^13,14^. In this pathway, chloromethane utilisation is initiated by a corrinoid- and tetrahydrofolate-dependent methyltransferase system encoded by *cmuA* and *cmuB* respectively^15^, whose expression is regulated by chloromethane^14,16^. The *cmuA* gene is strongly conserved in chloromethane-degrading strains^12^, and has been used as a biomarker gene in studies of chloromethane degradation in different environments^11,17,18^. Here, we explore the topic of the interrelationships of sources and sinks of chloromethane with the model system of the *A*. *thaliana* phyllosphere. We use *HOL1* and *cmuA* as biomolecular markers of chloromethane emission and consumption, respectively, to investigate the potential role of chloromethane-degrading bacteria as filters of plant emissions of chloromethane to the atmosphere.

## Results

A bipartite model laboratory system was set up consisting of well-studied organisms at the molecular level, *A*. *thaliana* for which the mechanisms of chloromethane production were identified, and *Methylobacterium extorquens* CM4, the best characterised chloromethane-degrading bacterium.

### Variants of *A*. *thaliana* with different expression of *HOL1*

The gene product of At2g43910 (*Arabidopsis* Information Resource, www.arabidopsis.org), designated *HOL1* for “Harmless to Ozone Layer”, was shown to be responsible for the major part of chloromethane emissions in *A*. *thaliana* ^8^. A T-DNA insertion mutant of *A*. *thaliana* wild-type (ecotype Col-0) causing a knock-out mutation in *HOL1* was obtained from the Salk Institute (http://signal.salk.edu) (Supplementary Information Fig. S1A). PCR genotyping of three *hol1* mutant lines (*hol1–1*, *hol1–2*, and *hol1–3*) allowed us to isolate homozygous *hol1-1* mutant plants (Supplementary Information Fig. S1B). Two lines of *A*. *thaliana* overexpressing *HOL1*, termed HOL1-OX1 and HOL1-OX6^19^, were also characterised with respect to *HOL1* expression. As evaluated by RT-qPCR, *A*. *thaliana hol1* plants showed significantly lower (*p* < 0.0003) expression of *HOL1* (20,000-fold). Conversely, significantly higher (*p* < 0.00003) expression of *HOL1* was observed in HOL1-OX1 ( > 27-fold) and HOL1-OX6 ( > 50-fold) plants compared to *A*. *thaliana* wild type (Supplementary Information Fig. S1C). Homozygous mutants *hol1-1* (lowest *HOL1* expression) and HOL1-OX6 (highest *HOL1* expression) were chosen for further analysis, and designated as *hol1* and HOL1-OX, respectively.

### Quantification of *cmuA* gene content and expression in the phyllosphere of *A*. *thaliana HOL1* variants

A quantitative PCR (qPCR) protocol targeting the chloromethane dehalogenase gene *cmuA* and the universal 16S ribosomal RNA gene in DNA obtained from washing of the leaf surface of *A*. *thaliana* was developed. Quantification of copy numbers of *cmuA* and 16S rRNA genes was achieved, with a lower limit of 15 and 750 copies for *cmuA* and 16S rRNA genes respectively (Supplementary Information Fig. S2).

**Figure 1:**
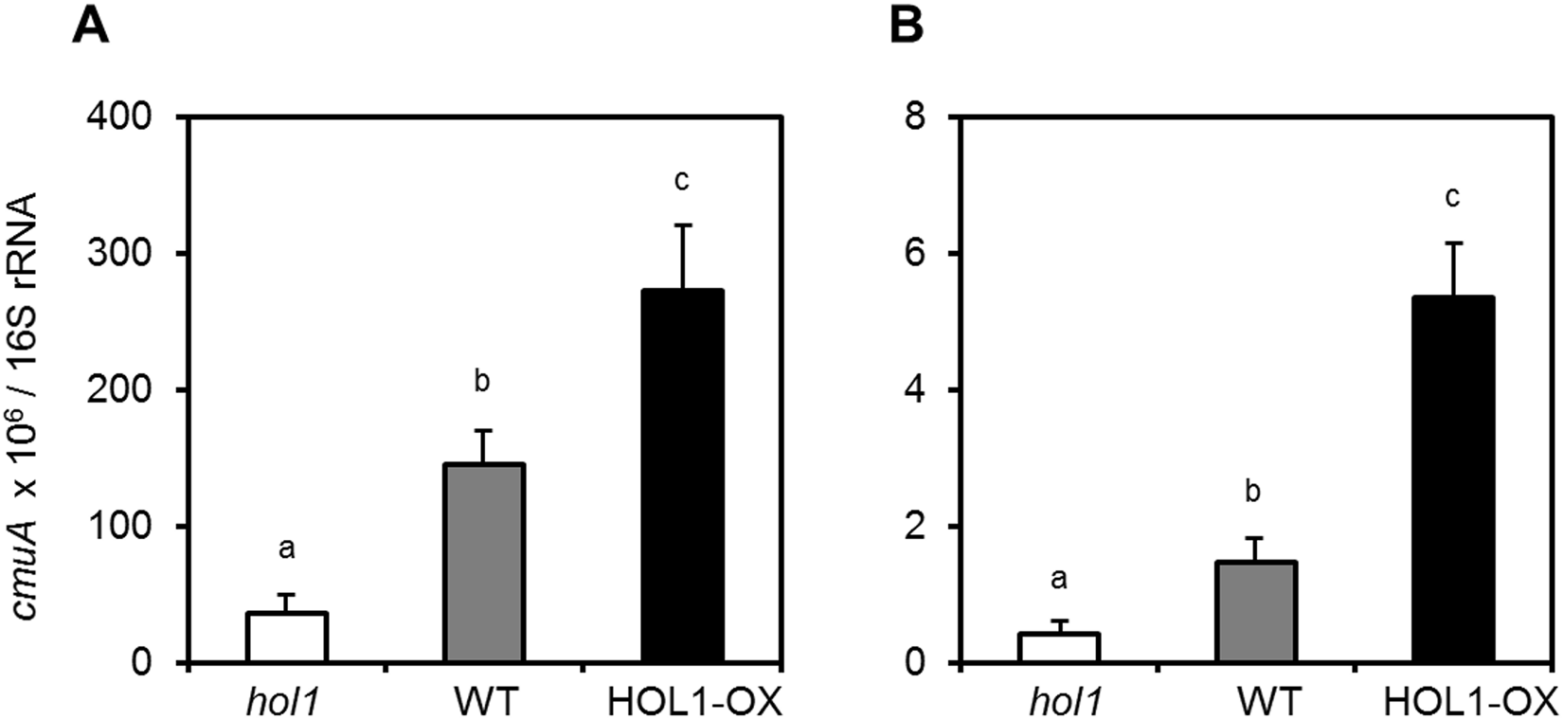
Relative abundance of *cmuA* in the phyllosphere of *A*. *thaliana HOL1* variants. qPCR was performed on DNA from leaf surface washings (**A**) and from total leaves (**B**). Results are expressed as copy number ratios of *cmuA* to 16S rRNA gene per µg of template DNA. Error bars represent propagated standard errors of three biological replicates, and small letters (a–c) indicate statistical significance at p < 0.05 by Student’s t-test.

The observed ratio of *cmuA*/16S rRNA gene copies was significantly higher (*p* = 0.049) in *A*. *thaliana* HOL1-OX plants, and significantly lower (*p* = 0.004) in *hol1* plants, as compared to wild type plants (Fig. 1A). Per weight, this corresponded to *cmuA* gene copy numbers per mg of fresh leaf of 13 ± 3, 71 ± 28 and 157 ± 43 on *hol1*, wild type and HOL1-OX plants respectively (Supplementary Information Table S1). In contrast, no significant differences of 16S rRNA gene copies per mg of fresh leaf (~10^5^ copies per mg, *p* > 0.4) were observed for *hol1*, wild type and HOL1-OX plants.

Similar trends were observed in experiments performed with total leaf DNA, i.e. DNA extracted from both the surface and the interior of leaves (Fig. 1B). The obtained *cmuA*/16S rRNA ratio was approximately 100-fold higher with leaf surface DNA than with leaf total DNA. The number of gene copies of the 16S rRNA gene per mg fresh leaf was much higher in leaf total DNA (4.9 × 10^5^ for leaf surface DNA and of 6.9 × 10^7^ for leaf total DNA, Table S1), likely because of high levels of plastid DNA containing this gene in DNA preparations.

**Figure 2:**
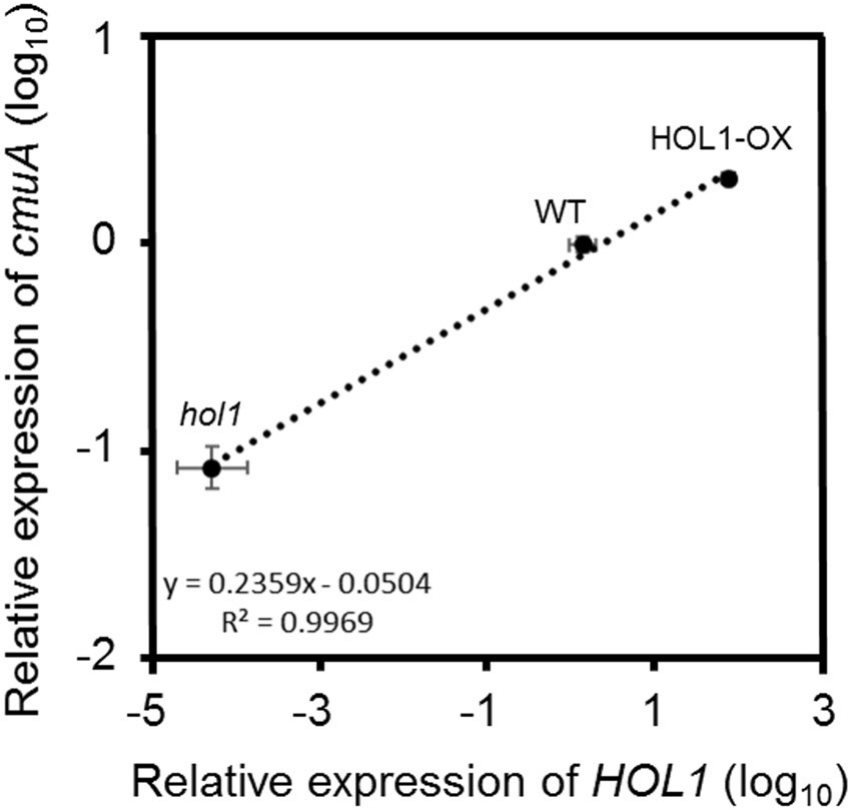
Relative expression of *cmuA* in the phyllosphere of *A*. *thaliana HOL1* variants. Relative expression levels of *HOL1* (in reference to *ACT2*) and *cmuA* (in reference to 16S rRNA) were evaluated by RT-qPCR on total RNA from the 3 variants (*hol1*, WT and HOL1-OX) of *A*. *thaliana*. Expression levels were computed relative to wild-type *A*. *thaliana* using the comparative threshold amplification cycle method (2^−ΔΔCt^)^20^. Average relative expression of *HOL1* is plotted against average relative expression of *cmuA* (three biological replicates). Dotted line shows the linear regression (R square = 0.997, p = 0.035). Error bars represent propagated errors associated with *cmuA* and *HOL1* expression.

The expression of *cmuA* gene relative to 16S rRNA gene was investigated by RT-qPCR on total RNA preparations from plant leaves. Under our experimental conditions, 16S rRNA fulfills criteria for a reference gene as described^20^. As observed at the DNA level, differences were observed between different variants of *A*. *thaliana* (Fig. 2), with HOL1-OX plants showing significantly higher expression of *cmuA* ( > 2-fold, *p = *0.00005) compared to wild type *A*. *thaliana*, and *hol1* plants showing 10 times lower (*p* = 0.00003) relative expression of *cmuA* compared to wild type *A*. *thaliana*. A significant and positive linear correlation (Pearson’s correlation coefficient = 0.998, p = 0.035 at 95% confidence interval) was found between the expression of both plant *HOL1* gene for chloromethane production and the bacterial gene *cmuA* for chloromethane degradation (Fig. 2).

### Diversity of bacteria in the *A*. *thaliana* phyllosphere

The correlation between *HOL1* genotype and *cmuA* gene presence and expression was investigated further. In the 3 *A*. *thaliana HOL1* variants, the composition and diversity of the phyllosphere microbiota was compared. Sequence analyses of 16S rRNA and *cmuA* gene fragments, obtained after PCR amplification from leaf surface DNA and pyrosequencing was performed (Tables 1 and 2, Supplementary Information Table S4). DNA yields were similar for the 3 variants, and the numbers of sequences obtained after PCR amplification were similar in all variants for the 16S rRNA gene. In contrast, numbers were different for the *cmuA* gene in the 3 variants, in good agreement with qPCR analysis (Supplementary Information Table S4). Based on 16S rRNA gene analysis (V1-V3 region), total microbiota was similar in the 3 variants. In total, 152 OTUs were detected with > 0.01 average abundance, 44 (28%) of which were common to the phyllospheres of the 3 variants, largely dominated by *Methylobacterium* and contributing to over 95% of the total sequences recovered (Table 1). The chloromethane-specific microbial subcommunity evidenced by analysis of the PCR-amplified *cmuA* gene showed limited sequence diversity (13 OTUs). Of these, 6 OTUs were closely similar to *cmuA* genes of known chloromethane-degrading strains.

**Table 1:** Most abundant bacterial genera (%) based on 16S rRNA sequence analysis in the phyllosphere of *A*. *thaliana* variants.

Genus^a^	WT	*hol1*	HOL1-OX
*Methylobacterium*	80.8 ± 3.8	89.4 ± 6.7	87.0 ± 4.2
*Sphingomonas*	6.5 ± 1.2	1.5 ± 1.7	5.0 ± 0.2
*Rhodococcus*	2.9 ± 2.9	2.3 ± 0.2	4.5 ± 3.2
*Nocardioides*	1.4 ± 0.8	0.7 ± 0.8	0.2 ± 0.2
*Sphingobium*	1.1 ± 0.8	0.4 ± 0.1	0.7 ± 0.1
*Pseudonocardia*	0.8 ± 0.3	0.4 ± 0.2	0.4 ± 0.1
TM7^b^	0.5 ± 0.3	0.4 ± 0.3	0.1 ± 0.0
*Aeromicrobium*	0.4 ± 0.2	0.4 ± 0.3	0.8 ± 0.6
*Microbacterium*	0.2 ± 0.1	0.6 ± 0.6	0.2 ± 0.2

^a^Genera with average abundance ≥ 0.5% in at least one of the 3 *A*. *thaliana* variants.

^b^All TM7-associated sequences were found within a single OTU.

**Table 2:** Diversity of the *cmuA* chloromethane dehalogenase gene in the phyllosphere of *A*. *thaliana* variants.

OTU	Closest homolog (accession number)	Sequence identity (%)		Recovered sequences^a^	
			WT	*hol1*	HOL1-OX
OTU1	*Methylobacterium extorquens* CM4 (AJ011316.1)	99.8	2454	1	16448
OTU2	*Hyphomicrobium* sp. SAC-1 (AJ871015.1)	98.8	459	1	3514
OTU3	*Hyphomicrobium* sp. MC1 (FN667867.2)	99.8	103	1	2100
OTU4	*Aminobacter ciceronei* IMB-1 (AF307143)	89.8	39	1	294
OTU6	*Aminobacter ciceronei* IMB-1 (AF307143)	89.3	30	205	0
OTU7	*Rhodobacteraceae bacterium* 198 (AJ810827.1)	99.3	21	0	234
OTU9	*Rhodobacteraceae bacterium* 179 (AJ810826.1)	99.5	0	0	121
OTU11	*Hyphomicrobium* sp. SAC-1 (AJ871015.1)	85.9	0	98	0
OTU19^b^	Uncultured marine bacterium (AJ810832.1)	91.0	0	7	14
OTU20	*Hyphomicrobium* sp. SAC-1 (AJ871015.1)	87.0	0	0	22
OTU22	*Hyphomicrobium* sp. SAC-1 (AJ871015.1)	87.0	22	0	0
OTU28^b^	Uncultured marine bacterium (AJ810832.1)	89.7	0	8	0
OTU35	*Aminobacter ciceronei* IMB-1 (AF307143)	99.3	0	0	17

^a^Numbers of sequences recovered by pyrosequencing after PCR amplification from the same amount of leaf wash DNA preparation, averaged across 6 samples (technical duplicates of biological triplicates) for each *A*. *thaliana* variant.

^b^Closest cultivable chloromethane degraders were *Hyphomicrobium* sp. MC1 for OTU19 (77.0% sequence identity) and *Methylobacterium extorquens* CM4 for OTU28 (78.2%), respectively.

### *In planta* detection of chloromethane production

**Figure 3:**
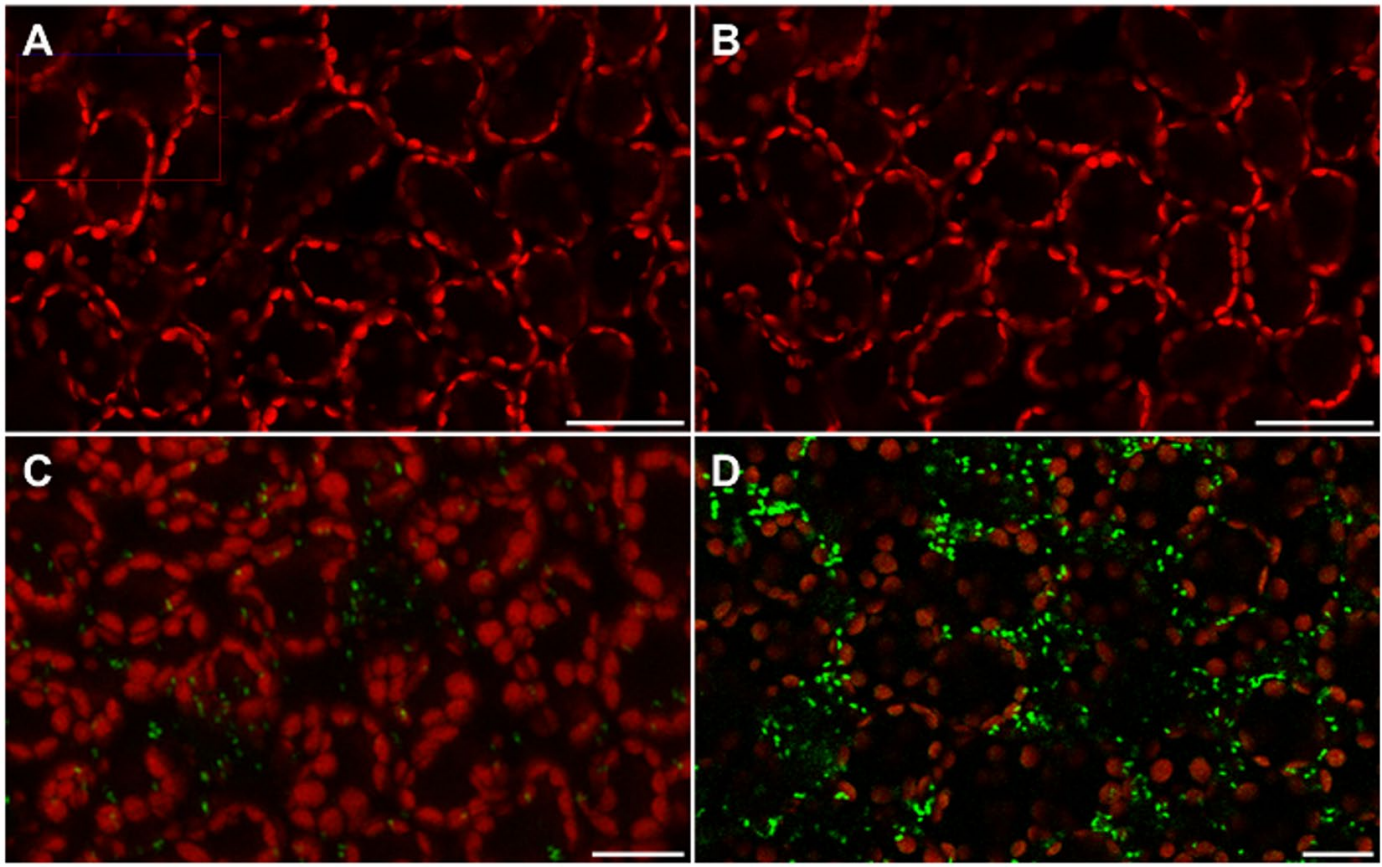
Confocal laser scanning micrographs showing *in planta* chloromethane dehalogenation. Yellow fluorescent bacteria (green spots) are visible on plant leaf surfaces along with autofluorescence (red) from chlorophyll in *A*. *thaliana hol1* (panel B), WT (panel C) and HOL1-OX (panel D) and an uninoculated control (panel A). Bars, 20 µm.

Detection of the amounts of chloromethane production by a bacterial fluorescent bioreporter, including on leaves of *A*. *thaliana*, was previously described^21^ (Fig. 3). This bioreporter consists of the chloromethane-degrading strain *M*. *extorquens* CM4 containing a plasmid harbouring the chloromethane dehalogenase *cmuA* gene promoter region fused upstream to the *syfp2* gene for yellow fluorescent protein (YFP)^22^. Fluorescence of the bioreporter strain was only observed in the presence of methyl halides, and was proportional to their concentration^21^. Here, two-weeks old seedlings of wild type, *hol1*, and HOL1-OX *A*. *thaliana* variants were sprayed with a bioreporter cell suspension, and YFP fluorescence was observed using confocal laser scanning microscopy after 24 h incubation (Fig. 3).

**Figure 4:**
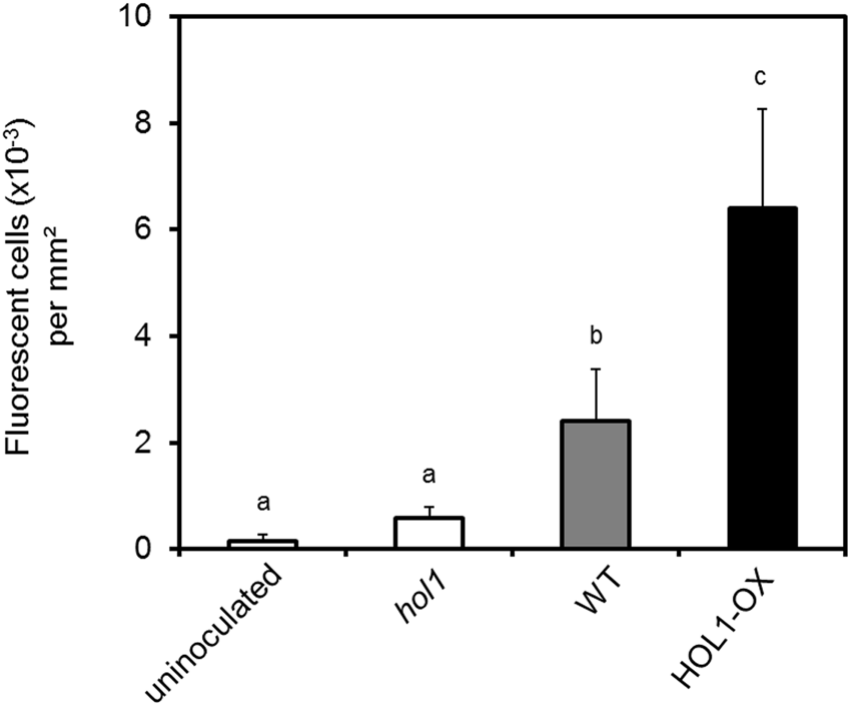
Quantification of fluorescent epiphytes on leaves inoculated with the live bacterial bioreporter. Images were taken by confocal laser scanning microscope after 24 hours of inoculation and analysed by ImageJ. Error bars represent the standard deviation of three biological replicates and small letters (**a**–**c**) shows statistical significance at p < 0.05 by Student’s t-test.

No fluorescent cells were observed on leaves of *hol1* plants, whereas a large number of fluorescent cells were visible on leaves of HOL1-OX and wild**-**type plants (Fig. 3). Quantification of fluorescent cells showed that the density of fluorescent cells on plant leaf surfaces was significantly higher for HOL1-OX plants than for wild type ( > 2.6-fold, *p = *0.02) and for *hol1* ( > 10-fold, *p = *0.003) plants (Fig. 4). In contrast, the number of bioreporter bacteria colonising the phyllosphere, evaluated by determining copy numbers of the s*yfp2* on plant leaf DNA by qPCR, was not significantly different in the different plant variants (Supplementary Information Table S2).

**Figure 5:**
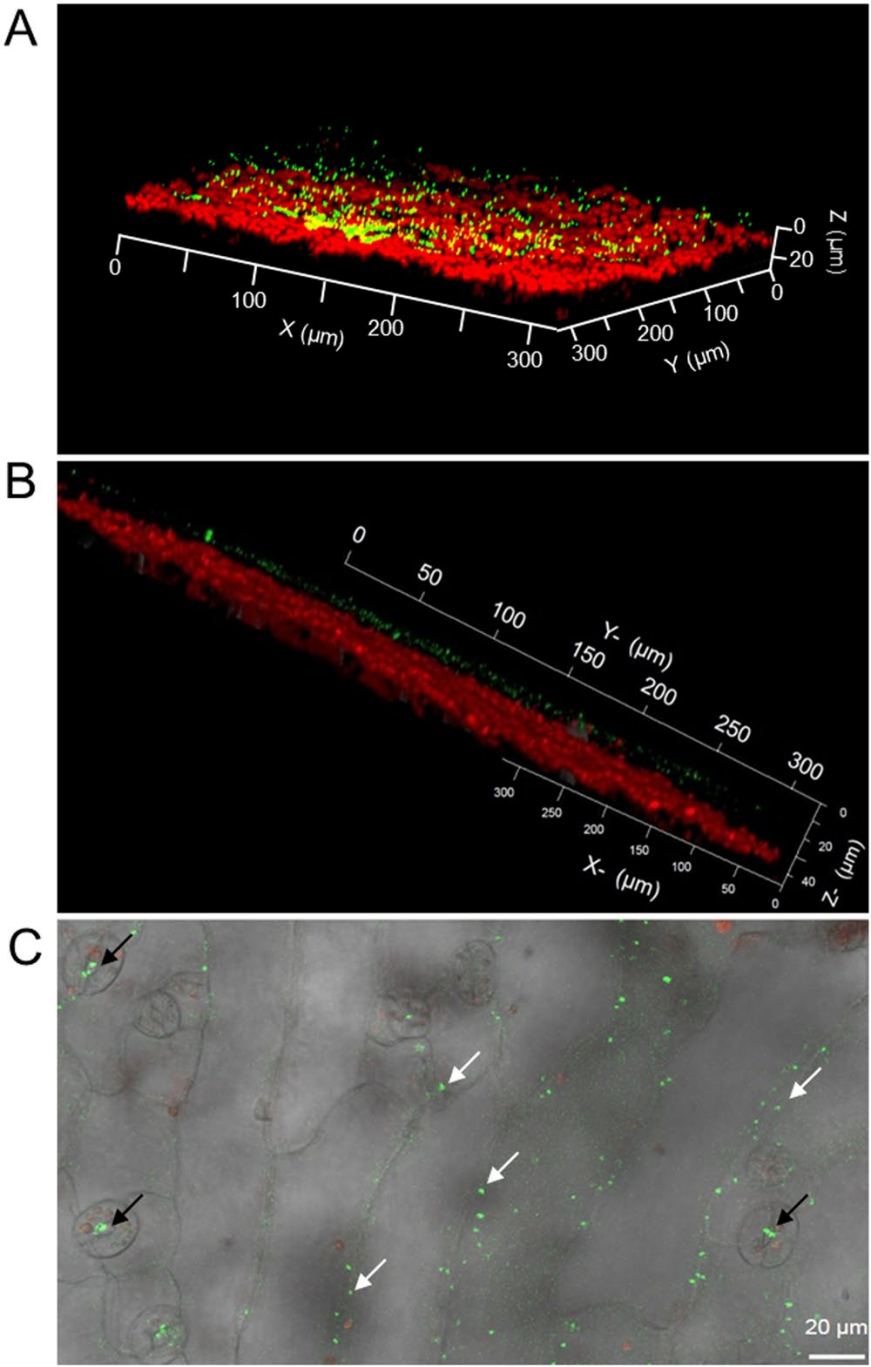
Localisation of bioreporter bacteria on the leaf surface of *A*. *thaliana*. (**A** and **B**) Representative confocal laser scanning microscope images (Z-stack mode to construct 3D image) showing yellow fluorescent chloromethane-bioreporter bacteria (green spots) on the surface of a wild type *A*. *thaliana* leaf, with autofluorescent chlorophyll (red) from plant cells underneath. (**C**) Phase contrast image merged to the corresponding fluorescence image shows fluorescent chloromethane-bioreporter bacteria (green spots), at stomata (black arrows), and the intercellular grooves of leaf epidermal cells (white arrows) of an inoculated wild-type plant.

Confocal microscopy also provided preliminary evidence on the source of chloromethane emissions on plant leaves (Fig. 5). Little is yet known on the localisation of the HOL1 methyltransferase and the expression profiles of the corresponding gene^23^. As reported previously for the tobacco phyllosphere with a methanol-based bioreporter^24^, the chloromethane-based bioreporter was frequently observed to be associated with stomata, where most gas exchange between plant leaves and the atmosphere takes place, as well as in the intercellular grooves and surfaces of epidermal cells (Fig. 5).

## Discussion

Bacterial chloromethane utilisation was previously investigated in several environments^18^, yet the phyllosphere environment, possibly the largest source of chloromethane^6^, had not yet been specifically explored in this regard. The model system introduced in this work allowed us to demonstrate that both gene copy number (Fig. 1) and gene expression (Fig. 2) of the bacterial *cmuA* gene for chloromethane dehalogenase in phyllospheric DNA and RNA correlate with expression levels of the gene *HOL1* responsible for chloromethane production (Supplementary Information Fig. S1C). In addition, application of a chloromethane-based bioreporter^21^ provided direct evidence for *in planta* expression of *cmuA* in the phyllosphere, and confirmed that *HOL1* gene variants of *A*. *thaliana* indeed emit chloromethane at different levels (Fig. 3). This finding is important because direct *in situ* real-time measurements of chloromethane production in the phyllosphere at the required sensitivity are difficult to achieve^21^.

Bacteria of the genera *Methylobacterium* and *Sphingomonas* are predominant colonisers of the phyllosphere^2,25^, as also found here (Table 1). Nevertheless, a predominance of *Methylobacterium* such as that observed here has not been reported so far, and may derive from controlled greenhouse conditions of our experiments compared to field conditions of previous studies. Indeed, site-specific factors were reported to strongly impact the proportion of *Methylobacterium* in a bacterial community^26^. *Sphingomonas*, the second most highly represented genus in our experiments, was substantially lower in the phyllosphere of the *hol1* variant (Table 1). *Sphingomonas* strains have been reported to protect *A*. *thaliana* against pathogenic bacteria^27^, and *A*. *thaliana* disrupted in the *HOL1* gene was more prone to pathogen attack, possibly due to reduced production of methyl thiocyanate (CH_3_SCN) from glucosinolate-derived thiocyanate by HOL1^28^. Indeed, disturbances in the metabolism of glucosinolate compounds was reported to modify the bacterial diversity in the rhizosphere of *A*. *thaliana* ^29^, and in the phyllosphere of vegetable plants^30^.

The proportion of chloromethane-degrading bacteria in the phyllosphere microbiota inferred from qPCR of the *cmuA* gene have some important implications. First, the total size of the bacterial community (as deduced from 16S rRNA gene copies) is similar for all three *A*. *thaliana* variants, and is higher by several orders of magnitude than those of chloromethane-degrading bacteria containing the *cmuA* gene (Supplementary Information Table S1). This is in keeping with well-established knowledge that carbon compounds such as methanol and sugars are released from plant leaves in much larger quantities than chloromethane^3,6^. Second, if one considers a single *cmuA* copy per chloromethane-degrading bacterium as usually observed so far^31^, the average number of chloromethane-degrading bacteria in the phyllosphere of wild-type *A*. *thaliana* may be estimated at approximately 71 ± 28 per mg of fresh leaf (Supplementary Information Table S1). Based on a chloromethane dehalogenase activity of 1,670 µmol per day per mg protein by *M*. *extorquens* CM4^32^, and on a value of approx. 165 fg protein per bacterial cell^33^, this number of bacteria could transform about 20 pmol chloromethane per day per mg of fresh leaf. By comparison, total emissions of chloromethane from wild type *A*. *thaliana* were reported at 0.6 pmol per day per mg of fresh leaf^8^, i.e. approximately an order of magnitude lower. This suggests not only that the phyllosphere microbiota can serve as an efficient filter for plant emissions of chloromethane, but also that chloromethane emissions from plants reported in the literature reflect the difference between chloromethane emissions from vegetation and degradation of chloromethane by vegetation-associated bacteria, rather than total chloromethane production by vegetation.

Despite widely available methanol, the modest amounts of chloromethane produced by *A*. *thaliana* are associated with an increased presence of chloromethane-degrading methylotrophic bacteria containing the *cmuA* gene in the phyllosphere. Whether this is solely due to chloromethane-dependent growth or to another as yet unknown phenomenon is still unknown, and deserves further investigation.

In conclusion, the biomolecular tools developed here to quantify gene signatures for chloromethane production and utilisation, as well as to detect and potentially quantify chloromethane production, may eventually contribute to better estimates of the overall budget of chloromethane crucial to environmental issues associated with destruction of stratospheric ozone. This, however, will require a better understanding of bacterial chloromethane utilisation in the environment. An important open question in this context is whether pathways for utilisation of chloromethane other than the *cmu* pathway, the only microbial pathway for chloromethane degradation characterised so far, are also relevant in defining the bacterial sink for natural emissions of chloromethane to the atmosphere, and from the phyllosphere in particular.

## Methods

### Chemicals and reagents

All chemicals and reagents (purity: > 99%) were obtained from Sigma-Aldrich unless otherwise stated. Buffers, culture media and solutions were prepared in ultra-pure water (PURELAB classic, ELGA) and sterilisation was done by autoclaving (20 min at 121 °C under 2 bars) or by filtration (0.2 µm, Nalgene).

### Bacterial strains and growth conditions

Chloromethane-degrading *Methylobacterium extorquens* CM4 (NCIMB 13688, VKM B-2223) and the bioreporter strain were laboratory stocks conserved at −80 °C in 10% glycerol in minimal mineral medium (M3)^14^. Liquid cultures were performed in 50 mL M3 medium in Erlenmeyer flasks (250 mL) fitted with sealed mininert valve caps (Supelco) and incubated at 30 °C with agitation at 100 rpm. Liquid M3 medium was supplemented with 20 mM methanol and/or chloromethane as described previously^12^. Growth of liquid cultures was monitored by measuring optical density at 600 nm.

### Plant material and growth conditions

All plants used in this study belonged to the *Arabidopsis thaliana* Columbia (Col-0) background. The Salk T-DNA lines of *hol1* mutant plants (SALK_005204, *hol1-1*, *hol1-2*, and *hol1-3*) were obtained from the Salk Institute (http://signal.salk.edu/cgi-bin/tdnaexpress), and seeds of the *A*. *thaliana* lines HOL1-OX1 and HOL1-OX6 (expressing *HOL1* under the control of the *CaMV 35 S* promoter) were obtained from Drs. Yukari Nagatoshi and Tatsuo Nakamura (Yokohama National University, Yokohama, Japan). Plants were grown at 22 °C with a 12 hours light period in standard horticultural soil in a plant growth chamber (PGC-15.5HILX) (Percival Scientific, USA). Relative humidity was 60% to 80%, and photon fluence rate from white fluorescent tubes was 60 μmol m^−2^ s^−1^ at the level of rosettes^34^.

Wild-type (Col-0) and *HOL1* gene mutants of *A*. *thaliana* were screened by PCR reactions (Supplementary Information Fig. S1).

### DNA extraction from plants

For leaf total DNA, fresh green leaves (2–3 leaves, 100–230 mg) were plucked from plants just before flower bolting stage. Total DNA from leaves was extracted using the FAST DNA spin kit (MP Biomedicals, Santa Ana, CA) as described^35^. Leaf material was transferred to lysing matrix A tubes and macerated in 200 µL of phosphate buffer (120 mM, pH 8). After addition of 600 µL CLS-VF buffer and 200 µL PPS buffer from the kit, leaf material was lysed using a Mikro-Dismembrator (Sartorius Stedim Biotech, France) with 3 treatments of 1 min each at 3000 min^−1^. DNA was then purified from the lysate according to the manufacturer’s protocol. Final elution of purified DNA was performed in 100 µL of DNase-free sterile water. DNA was quantified by NanoDrop and purity was checked by gel electrophoresis on agarose gel (1%).

Leaf surface bacterial DNA was extracted from *A*. *thaliana* leaf washings using a protocol adapted from the literature^36,37^. Briefly, 10–15 leaves of *A*. *thaliana* plants (1–2 g fresh weight) were washed in 50 mL sterile tubes containing 30 mL TE buffer (pH 7.5) with 0.2% Silwet L-77 (GE Bayer Silicones). Bacteria were dislodged from the leaf surface by shaking (350 rpm, 10 min) and sonication (25% amplitude, 30 seconds, Vibracell 75042, Bioblock Scientific), followed by vortexing for 30 seconds. The resulting suspensions were then filtered (Nylon net filters 180 µm; Millipore) to separate leaf material from cell suspensions. Percoll was added to the cell suspensions, and centrifuged at 12′000 *g* for 10 min. Supernatant was removed, and the obtained cell pellet was washed in TE buffer (pH 7.5), resuspended in 200 µL of TE buffer (pH 7.5), and transferred to lysing matrix A tubes for DNA extraction using the Fast DNA spin kit (MP Biomedicals, Santa Ana, CA). Cells lysis was performed in 800 µL of CLS-VC reagent from the kit using Mikro-Dismembrator (Sartorius Stedim Biotech, France) with three treatments of 45 s each at 3000 min^−1^. DNA was extracted from the lysate using the protocol as described above for leaf total DNA.

### Total RNA extraction from plants

Total RNA from plant leaves was extracted from 8–10 weeks old wild-type, *hol1* and HOL1-OX6 plants of *A*. *thaliana*. Leaf samples (100 mg) were transferred to 1.5 mL RNase-free tube and immediately flash frozen in liquid nitrogen. The leaf material was lysed under liquid nitrogen in lysing matrix A tubes (MP Biomedicals, Santa Ana, CA) using Mikro-Dismembrator (Sartorius Stedim Biotech, France) with 3 treatments of 1 min each at 3000 min^−1^. Total RNA was extracted using the NucleoSpin RNA plant kit (Macherey-Nagel, France), according to the manufacturer’s protocol. RNA was quantified by NanoDrop, and purity was checked by gel electrophoresis (1% agarose gel). Absence of DNA contamination in the RNA was checked by PCR with *ACT2* gene primers (Supplementary Information Table S3).

### Primer design

Primers used in this study are listed in Supplementary Information Table S3. Primers for qPCR of *HOL1* plant gene were designed using PrimerQuest tool (Integrated DNA Technologies), and their specificity was verified with a BLAST analysis at NCBI. Primers for qPCR of *cmuA* were designed on the basis of alignments of *cmuA* sequences from reference chloromethane-degrading strains. Already described forward primer cmuA802F^17^ and a newly designed reverse primer cmuA968R with identity > 80% over the entire length of primer with all *cmuA* sequences in the NCBI database, were used.

### Quantitative PCR (qPCR)

Evaluation of *cmuA* and 16S rRNA gene copy number was performed by qPCR using GeneAmp 5700 Sequence Detection System (Applied Biosystems). Analyses were carried out in a 96 well reaction PCR plate (Applied Biosystems), using gene specific primers (Supplementary Information Table S3 for sequences and references of all primers) in a total reaction volume of 20 µL, as described previously^12^. Conditions for qPCR reaction consisted of initial denaturation at 95 °C for 10 min, followed by 40 cycles of denaturation at 95 C for 15 s and annealing/elongation at 60 °C for 60 s. Negative controls without DNA and control reactions to check for absence of inhibition of PCR amplification in each DNA sample were performed by spiking known quantities of *M*. *extorquens* CM4 DNA. Specificity of amplification was determined after qPCR cycles from dissociation curves obtained by increasing 1 °C per 30 s from 60 °C to 90 °C, or after gel electrophoresis of PCR products on 2% agarose. The gene copy number of *cmuA* and 16S rDNA per µg of phyllosphere DNA were determined using calibration curves obtained from qPCR of tenfold dilution series of DNA standards (Supplementary Information Fig. S2). Analyses were performed with at least three biological replicates of each plant type and three technical repeats for each biological sample.

### Quantitative reverse transcriptase – PCR (RT-qPCR)

Expression of *HOL1* gene in plant leaves was quantified using *ACT2* as a reference gene in RT-qPCR analysis. Superscript III Reverse Transcriptase Kit (Invitrogen) was used to synthesize cDNA from plant total RNA (500 ng) using random primers (Roche) according to the manufacturer’s protocol. Expression of bacterial *cmuA* gene in plant leaves was also quantified by RT-qPCR analysis, using 16S rRNA as a reference gene. Phyllosphere total RNA (500 ng) was used to synthesize cDNA using Superscript III Reverse Transcriptase Kit (Invitrogen), and gene specific primers (cmuA968R and PROK1492R for *cmuA* and 16S rRNA respectively) according to the manufacturer’s protocol. After the RT reaction, the cDNA was diluted 2.5 times, and 5 µL used as template for qPCR. Real-time qPCR measurements were performed as described above for DNA samples. Control reactions without RT enzyme were performed for each RNA extraction to check for absence of contaminating DNA. The relative level of *cmuA* and *HOL1* gene expression was estimated using the comparative threshold amplification cycle method (2^−ΔΔCt^) as described^20^. Analysis was performed with three to five biological replicates of each plant type, with three technical repeats for each biological replicate.

### Pyrosequencing analysis

DNA was extracted as described above. Bacterial tag-encoded FLX 454 pyrosequencing of phyllosphere DNA (bTEFAP) was performed by Research and Testing Laboratory (Lubbock, TX, USA) as described previously^38–40^, with a Genome Sequencer FLX instrument and Titanium protocols and reagents (Roche Applied Science, Indianapolis, IN). Primers Gray28F and Gray519R (Supplementary Information Table S4) spanning the variable regions V1–V3 in the 16S rRNA gene^38,39^ were used in a single step PCR reaction (35 cycles) using HotStar HiFidelity Polymerase (Qiagen, Valencia, CA). Primer cmuA802F^17^ was used together with primer cmuA1244R (Supplementary Information Table S4) for amplification of a 443 bp *cmuA* amplicon under the same conditions.

### Sequence processing and analysis

For amplicons of the 16S rRNA gene, obtained sequences were screened, failed sequence reads, low quality sequence ends, tags and primers were removed, and non-bacterial ribosome sequences, chimeras detected using black box chimera check software B2C2^41^, and short reads (<250 bp) were discarded as described previously^38–40^. Operational taxonomic units (OTUs) were assigned at 5% dissimilarity, commonly used to represent the genus level^42^, validated using taxonomic distance methods, and data reduction analysis performed as described previously^38,40^.

For *cmuA* amplicon analysis, Mothur^43^ was used to extract flow grams from sff files. Flow grams were de-noised and translated to DNA sequences, and reads with errors in the barcode or primer region, ambiguous bases, or homopolymer runs > 6 bp were removed. Quality filtering and sizing of reads (between 400 and 450 bp), conversion to fasta format, read dereplication, abundance sorting and removal of singletons was done with USEARCH^44^. Chimeras were filtered out using UCHIME within USEARCH. Iterative clustering of OTUs was carried out with UPARSE within USEARCH to a cutoff of 85%. The most abundant sequence within each OTU was chosen as representative of the OTU, and taxonomic affiliation was performed by Blast comparisons to the Genbank database.

### Inoculation of bioreporter to plant leaves

The bacterial bioreporter strain constructed based on plasmid harbouring the *syfp2* gene coding for yellow fluorescent protein (YFP) fused with the promoter region of *cmuA* gene^21^ was grown until mid-exponential phase (OD_600_ ~0.4) in M3 medium supplemented with MeOH (20 mM). Cells were harvested after centrifugation at 4500 x g, washed and resuspended in M3 medium at a final OD_600_ of 0.2.

*A*. *thaliana* wild type, *hol1* and HOL1-OX plants were grown in Petri dishes on Murashige and Skoog (MS) medium including vitamins (Duchefa) supplemented with 1% sugar and 0.7% pastagar. Plant seeds were surface sterilised (with 25% commercial solution of sodium hypochlorite for 10 minutes and then rinsed five times with sterile water) before sowing to avoid undesired bacteria on plant leaves. Plants were grown at 22 °C with 12 hours light period for 14-days in a plant growth room. Cells suspensions (5 mL per Petri dish containing 20 plants) were sprinkled uniformly on leaves and left to dry for 1 hour under a laminar flow hood. Petri dishes were then further incubated for 24 hours under the same plant growth conditions before observation under confocal microscopy.

### Confocal microscopy and image analysis

Leaves of similar sizes were cut from plants treated with the bioreporter, mounted on microscope glass slides and visualised for YFP fluorescence (YFP filter, 488 nm) under the 20x lens of confocal laser scanning microscope (Zeiss LSM710), and images were recorded. Three images from different locations per plant, and three plants for each genotype were randomly selected. Images were analysed to quantify YFP fluorescence on leaves using ImageJ software (http://rsbweb.nih.gov/ij/index.html). Fluorescence intensity was used to discriminate bacteria from the background. Each fluorescent spot identified by ‘Find maxima imageJ’ tool with noise value set at 50 was considered as a fluorescent bacterial bioreporter cell, and the number of fluorescent cells per mm² was measured. After confocal observation, total DNA was also extracted from inoculated leaves to confirm the equal abundance of bioreporter bacteria on each type of plant by qPCR of the *syfp2* gene.

### Statistical analysis

Experiments were performed in at least three biological replicates, with two to three technical repeats for each biological replicate. For statistical significance, data were analysed using Student’s t test, with different letters in figures indicating statistically significant differences at *p* < 0.05. Pearson’s correlation coefficient R for average relative expression levels of *HOL1* versus the average relative expression levels of *cmuA* (Fig. 2), and propagated errors, were calculated from errors associated with values of *cmuA* and *HOL1* expression based on ± 1 standard deviation of the mean of 3 replicate measurements^45^.

### Data Availability

The sequence data generated in this study were deposited to Genbank under accession numbers SAMN07314135-SAMN07314170 (Bioproject PRJNA393055).

## Electronic supplementary material

Supplementary Information
