# Supplementary material for: Correlated production and consumption of chloromethane in the Arabidopsis thaliana phyllosphere
Source: Sci Rep. 2017 Dec 14;7:17589. doi: 10.1038/s41598-017-17421-y (PMC5730606; doi:10.1038/s41598-017-17421-y)
Supplement: Supplementary file 1 — Supplementary Information [file 41598_2017_17421_MOESM1_ESM.pdf]

Supplementary Information

Correlated production and consumption of chloromethane in the *Arabidopsis thaliana* phyllosphere

Muhammad Farhan Ul Haque[1‡], Ludovic Besaury[1§], Thierry Nadalig[1], Françoise Bringel[1], Jérôme Mutterer[2], Hubert Schaller[2] and Stéphane Vuilleumier[1*]

Authors and affiliations

[1] Université de Strasbourg, CNRS, GMGM UMR 7156, Department of Microbiology, Genomics and the Environment, Strasbourg, France

[2] Institut de Biologie Moléculaire des Plantes, UPR2357 CNRS, Strasbourg, France

[‡] Present address: School of Environmental Sciences, University of East Anglia, Norwich, UK

[§] Present address: Université de Reims Champagne Ardenne, INRA, FARE UMR A614, Reims, France

[*] **Corresponding author**:

Stéphane Vuilleumier, Université de Strasbourg, Equipe Adaptations et Interactions Microbiennes dans l'Environnement, Département Microorganismes, Génomes, Environnement, UMR 7156 UNISTRA - CNRS, 28 rue Goethe, F-67000 Strasbourg, France, phone: +33-3-68-85-2022; fax: +33-3-68-85-1926; e-mail: vuilleumier@unistra.fr

Supplementary Information

HOL1 genotyping of plants

Wild-type (Col-0) and *HOL1* gene variants of *A. thaliana* were screened by performing two sets of PCR reactions. The first PCR was performed using gene specific primer pair (hol1 and hol2, Table S3) to confirm homozygosity of mutants. The second PCR was performed using primer hol1 and T-DNA specific primer jmlb2 (Supplementary Table S1) to confirm T-DNA insertion. Each PCR reaction mix (25 µL) contained 2.5 µL PCR buffer 10x (Tris-HCl 10 mM at pH 9.0, KCl 50 mM, $MgCl_2$ 1.5 mM, Triton 0.1% and BSA 0.2 mg mL^{-}), 0.4 µM of each primer, 0.12 µM of each dNTPs, 0.2 µL Taq DNA polymerase (5 U $µL^{-}$, Q biogene) and ultrapure filtered sterilised water. The PCR program involved initial denaturation at 95°C for 3 mins, followed by 35 cycles of three steps (94°C for 1 min, 60°C for 1 min and 72°C for 45 seconds), and final elongation at 72°C for 3 mins. PCR products were visualised under UV light using a Geldoc apparatus (Bio-Rad) after agarose gel electrophoresis.

Gene copy number calculation

Calibration curves were obtained to determine the gene copy number of *cmuA* and 16S rDNA per µg of template in the phyllosphere using tenfold dilution series of standards (10 ng to 10 fg, Supplementary Fig. S1) of *M. extorquens* CM4 genomic DNA. *M. extorquens* CM4 genome contain 5 copies of the 16S rRNA gene and 1 copy of the *cmuA* gene [1]. Assuming average molecular masses of 650 Daltons (Da) for a nucleotide pair of double-stranded DNA, gene copy number for a given quantity of standard for the calibration curve was calculated by [quantity (ng) / L (bp) x 650 (Da) x 10^{-9}] x 6.02 x 10^{23} x [copy number of gene per genome] where L is the sequence length (6180732 bp for *M. extorquens* CM4 strain). To calculate *syfp2* copy number, plasmid pME8266 (6381 bp, with 1 copy of *syfp2*) [2] was used as the standard. Threshold cycle (Ct) values were plotted against

copy number of serially diluted DNA standards, and the linear relationship obtained was used to calculate the number of gene copies per µg of phyllosphere DNA template in qPCR.

Supplementary Figures

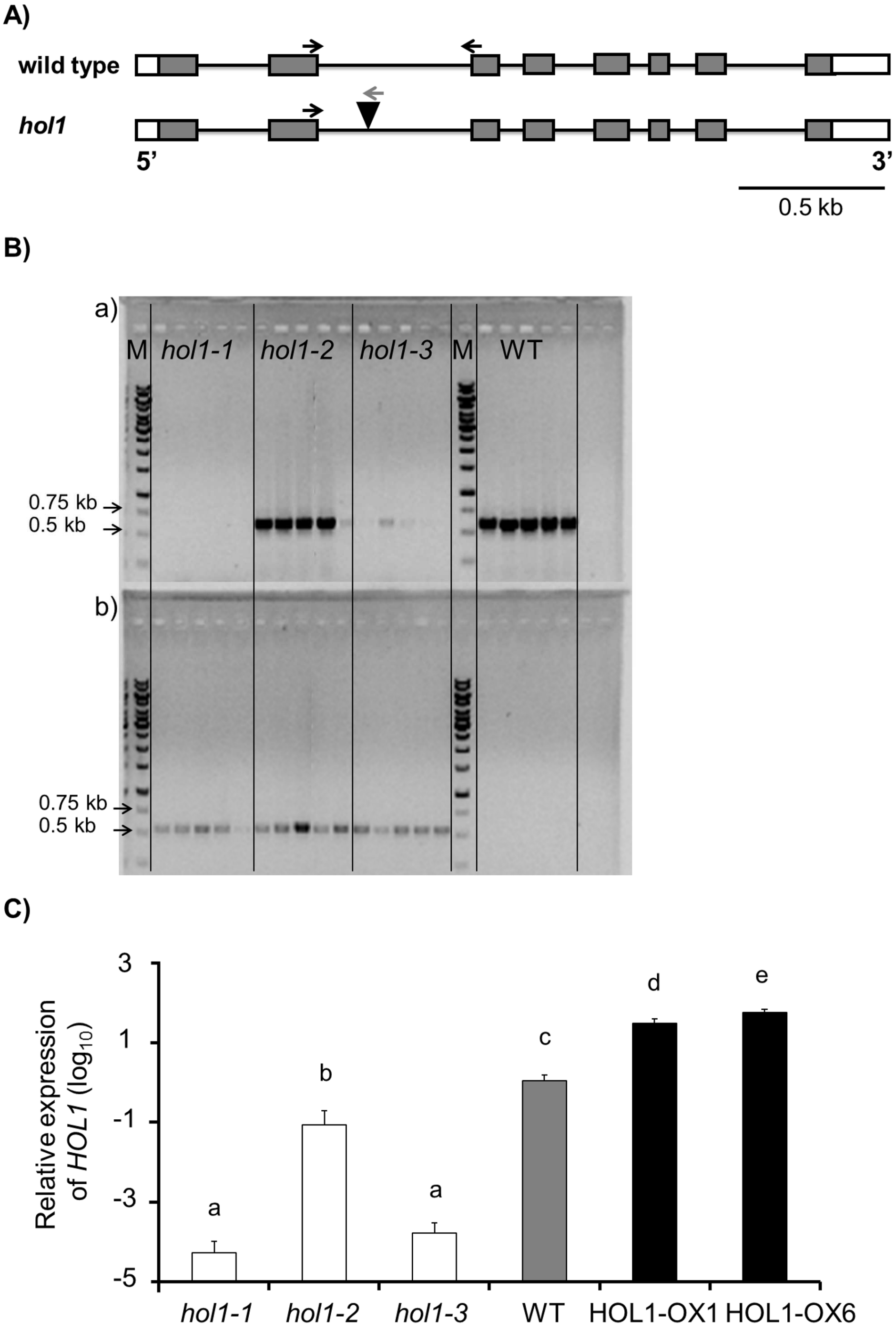

Fig. S1. Screening of *HOL1* variants of *A. thaliana*.

A) Organisation of gene *HOL1* in *A. thaliana* wild type (Col-0), based on locus data from the *Arabidopsis* Information Resource (http://www.arabidopsis.org). Gene *HOL1* comprises 8 exons (boxes) and 7 introns (lines) and encodes a 227- amino acids methyltransferase protein. The T-DNA

site of insertion in *hol1* mutant plants is shown (black triangle). Position of wild-type sequence hol1 and hol2 primers (black) and T-DNA primer jmlb2 (grey) used for genotyping are shown (see Table S3 for primer sequences).

B) Genotyping of gene *HOL1* in *A. thaliana* wild type (Col-0) and three independent lines (*hol1-1*, *hol1-2* and *hol1-3*) of *hol1* mutant plants using wild type primer pair hol1-hol2 (a) and T-DNA primer pair hol1-jmlb2 (b). M, marker line.

C) Relative expression of gene *HOL1* in wild-type *A. thaliana hol1* mutant lines, and *HOL1* overexpressor lines (HOL1-OX1 and HOL1-OX6), as measured by qRT-PCR (see Materials and Methods for calculation). Error bars represent the standard deviation of at least five biological replicates, and small letters (a – e) show statistical significance at $p < 0.05$ by Student's t-test. The 2 *A. thaliana* lines with lowest (*hol1-1*) and highest (HOL1-OX6) expression of *HOL1* were selected for subsequent experiments.

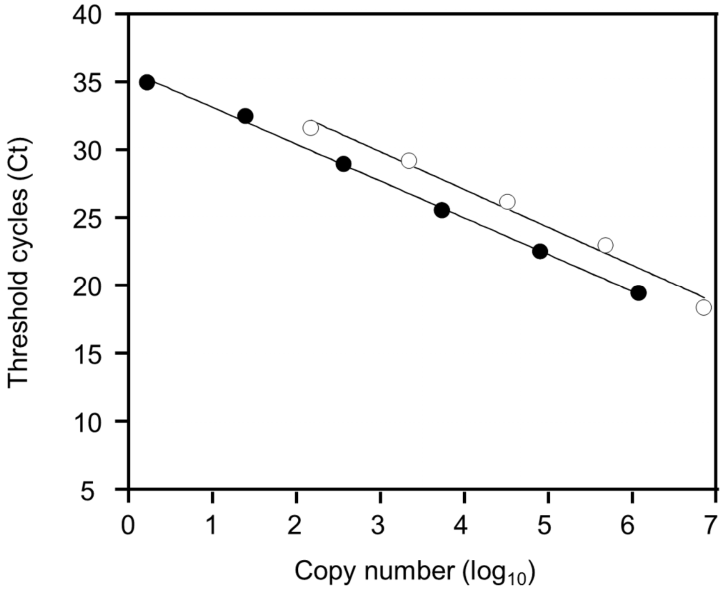

Fig. S2. Calibration curve for 16S rRNA and *cmuA* genes. Serial tenfold dilutions of standards (10 ng – 1 fg) of *M. extorquens* CM4 total DNA were used as template for qPCR, and the results expressed with DNA as gene copies against threshold cycle (Ct) values. Linear regression lines are y = 41.206 – 3.225x (R=0.999) and y = 38.759 – 3.136x (R=0.992) for 16S rRNA (white) and *cmuA* (black) respectively.

Supplementary Tables

Table S1. *cmuA* and 16S rRNA gene copies per mg of fresh leaf as measured by qPCR.

	cmuA	**16S rRNA**
**Leaf surface DNA**		
hol1	13.5 (± 3.2)	7.0 (± 5.6) · 10^{5}
WT	70.5 (± 27.6)	4.9 (± 0.4) · 10^{5}
HOL1-OX	157.2 (± 43.0)	6.2 (± 2.4) · 10^{5}
**Leaf total DNA**		
hol1	22.2 (± 15.9)	4.9 (± 0.7) · 10^{7}
WT	85.8 (± 9.6)	6.9 (± 3.1) · 10^{7}
HOL1-OX	164.7 (± 36.1)	3.2 (± 0.5) · 10^{7}

Table S2. ***syfp2*** **gene copies per mg of fresh leaves inoculated with bioreporter as measured by qPCR.**

Plant type	***syfp2*** **copies per mg of fresh leaves**
hol1	$5.3 (\pm 0.2) \cdot 10^{5}$
WT	$4.4 (\pm 0.2) \cdot 10^{5}$
HOL1-OX	$6.1 (\pm 0.3) \cdot 10^{5}$
not inoculated	$1.2 (\pm 0.6) \cdot 10^{0}$

Table S3. Primers used in the study

Target (purpose)		Primer sequence 5' – 3' [a]	Reference
Primers for bacterial genes			
cmuA	cmuA802F	TTCAACGGCGAYATGTATCCYGG	[3]
(qPCR)	cmuA968R	CCRCCRTTRTAVCCVACYTC	[4]
16S rRNA	Bact1369F	CGGTGAATACGTTCYCGG	[5]
(qPCR)	Prok1492R	GGWTACCTTGTTACGACTT	[5]
syfp2	MF34	ACAAGCAGAAGAACGGCATC	[2]
(qPCR)	MF35	GCTTGGACTGGTAGCTCAGG	[2]
16S rRNA	Gray28F	GAGTTTGATCNTGGCTCAG	[6]
(454)	Gray519R	GTNTTACNGCGGCKGCTG	[7]
cmuA	cmuA802F	TTCAACGGCGAYATGTATCCYGG	[3]
(454)	cmuA1244R	TABTCCATDATGGCYTCGAC	this study
Primers for plant genes			
HOL1	hol1	GGAGTCAGTCTTCTTCTAGCTTACC	[8]
(genotyping)	hol2	GTGCGCTTTCGGAAATATCCAATCC	[8]
T-DNA insertion	jmbl2	TTGGGTGATGGTTCACGTAGTGGG	[8]
HOL1	holqPCR_F	GAAAGCGCACTCGCGAAAGCTAAT	this study
(qPCR)	holqPCR_R	ATGCAGGTCTCATCTCCGGTTCAA	this study
ACTIN2	SS77	TTCAATGTCCCTGCCATGTATG	[9]
(qPCR)	SS78	AATACCGGTTGTACGACCAC	[99]

[a] Equimolar mixtures at degenerate positions: N (G,A,T,C); V (G,A,C); D (G,A,T); B (G,T,C); R (G,A); K (G,T); W (A,T); Y (T,C).

Table S4. Pyrosequencing analysis data

**Plant *HOL1* gene variant**	**WT**			***hol1***			**HOL1-OX**		
Biological triplicates	1	2	3	1	2	3	1	2	3
DNA concentration (ng/ml)	20.0	25.8	20.0	11.7	10.7	14.7	16.4	15.1	18.5
16S rRNA sequences[1]	11209/9791	8512/4502	12023/6677	16964/7859	3863/5856	4816/8255	12202/9540	7690/18562	12877/13528
cmuA sequences[1]	1593/1799	1436/716	263/445	0/0	410/5	0/225	747/22616	668/5161	1803/14526

[1] Total sequences retrieved in 2 sequencing runs on DNA from the same biological replicate

References

1 Marx, C. J. *et al.* Complete genome sequences of six strains of the genus *Methylobacterium*. *J. Bacteriol.* **194**, 4746-4748 (2012).

2 Farhan Ul Haque, M., Nadalig, T., Bringel, F., Schaller, H. & Vuilleumier, S. Fluorescence-based bacterial bioreporter for specific detection of methyl halide emissions in the environment. *Appl. Environ. Microbiol.* **79**, 6561-6567 (2013).

3 Miller, L. G. *et al.* Degradation of methyl bromide and methyl chloride in soil microcosms: Use of stable C isotope fractionation and stable isotope probing to identify reactions and the responsible microorganisms. *Geochim. Cosmochim. Acta* **68**, 3271-3283 (2004).

4 Nadalig, T. *et al.* Detection and isolation of chloromethane-degrading bacteria from the *Arabidopsis thaliana* phyllosphere, and characterization of chloromethane utilisation genes. *FEMS Microbiol. Ecol.* **77**, 438-448 (2011).

5 Suzuki, M. T., Taylor, L. T. & DeLong, E. F. Quantitative Analysis of Small-Subunit rRNA Genes in Mixed Microbial Populations via 5′-Nuclease Assays. *Appl. Environmental Microbiol.* **66**, 4605-4614 (2000).

6 Handl, S., Dowd, S. E., Garcia-Mazcorro, J. F., Steiner, J. M. & Suchodolski, J. S. Massive parallel 16S rRNA gene pyrosequencing reveals highly diverse fecal bacterial and fungal communities in healthy dogs and cats. *FEMS Microbiol. Ecol.* **76**, 301-310 (2011).

7 Ishak, H. D. *et al.* Bacterial diversity in *Solenopsis invicta* and *Solenopsis geminata* ant colonies characterized by 16S amplicon 454 pyrosequencing. *Microb. Ecol.* **61**, 821-831 (2011).

8 Rhew, R. C., Ostergaard, L., Saltzman, E. S. & Yanofsky, M. F. Genetic control of methyl halide production in *Arabidopsis*. *Current Biol.* **13**, 1809-1813 (2003).

9 Bouvier-Navé, P. *et al.* Involvement of the phospholipid sterol acyltransferase1 in plant sterol homeostasis and leaf senescence. *Plant Physiology* **152**, 107-119 (2010).
